# Neuroanatomical bases of effortful control: evidence from a large sample of young healthy adults using voxel-based morphometry

**DOI:** 10.1038/srep31231

**Published:** 2016-08-09

**Authors:** Rui Nouchi, Hikaru Takeuchi, Yasuyuki Taki, Atsushi Sekiguchi, Yuka Kotozaki, Seishu Nakagawa, Carlos Makoto Miyauchi, Kunio Iizuka, Ryoichi Yokoyama, Takamitsu Shinada, Yuki Yamamoto, Sugiko Hanawa, Tsuyoshi Araki, Yuko Sassa, Ryuta Kawashima

**Affiliations:** 1 Creative Interdisciplinary Research Division, Frontier Research Institute for Interdisciplinary Science (FRIS), Tohoku University, Seiryo-machi 4-1, Sendai 980-8575, Japan; 2 Smart Ageing International Research Center, Institute of Development, Aging and Cancer, Tohoku University, Sendai, Japan; 3 Division of Developmental Cognitive Neuroscience, Institute of Development, Aging and Cancer, Tohoku University, Sendai, Japan; 4 Department of Nuclear Medicine and Radiology, Institute of Development, Aging and Cancer, Tohoku University, Sendai, Japan; 5 Division of Medical Neuroimaging Analysis, Department of Community Medical Supports, Tohoku Medical Megabank Organization, Tohoku University, Sendai, Japan; 6 Department of Adult Mental Health, National Institute of Mental Health, National Center of Neurology and Psychiatry, 4-1-1 Ogawa-Higashi, Kodaira, Tokyo 187-8553, Japan; 7 Department of Functional Brain Imaging, Institute of Development, Aging and Cancer, Tohoku University, Sendai, Japan; 8 Graduate Schools for Law and Politics, The University of Tokyo, Tokyo, Japan; 9 Department of Psychiatry, Tohoku University Graduate School of Medicine, Sendai, Japan; 10 Japan Society for the Promotion of Science, Tokyo, Japan; 11 Faculty of Medicine, Kobe University, 7-5-1kusunoki-cho, Kobe, 950-0017 Japan

## Abstract

Effortful control (EC) is a base of individuality in cognition and psychological adjustment. EC is defined as a capacity to control responses and behaviors. We investigated associations between individual differences of EC and regional gray and white matter volume (rGMV/rGMV) in 374 men and 306 women (age, 20.61 ± 1.82 years) using Japanese version of Effortful control scale (J-ECS). J-ECS consists of three subscales such as inhibitory control (IC), activation control (ACTC), and attentional control (ATC). Results showed that (a) IC was associated with larger rGMV in the dorsal part of anterior cingulate cortex (dACC), the supplementary motor area (SMA) and the pre SMA and larger rWMV in the dACC, (b) ACTC was correlated with smaller rGMV in the insula and the putamen, and (c) ATC was associated with larger rWMV in the inferior frontal gyrus, orbital frontal gyrus, ACC, and insula. Our study revealed key neuroanatomical correlations between EC and rGMV and rWMV.

Effortful control (EC) is “the efficiency of executive attention, including the ability to inhibit a dominant response and to activate a subdominant response, to plan, and to detect errors (p.129)^1^”. In fact, the control of behaviors and cognitive processes such as attention^2^ is a base for successful emotional regulations and control of thoughts and actions^1^,^3^. Actually, EC plays a key role in self-regulation. Self-regulation is an ability to manage goal-directed activities across time and contexts^4^. Thus, EC is central to adaptive functioning. Previous studies have indicated associations between EC and psychological adjustments. For example, the EC score was negatively correlated with clinical symptoms such as social anxiety and depression^5^,^6^. The EC was positively correlated with subjective wellbeing^6^. Additionally, high EC is apparently a protective factor against compulsive buying and some disorder symptoms (eating disorder and personality disorders)^7^,^8^. The evidence indicates that the EC has critical role in shaping psychological and social adjustment^6^.

Although EC is conceptualized as a critical component of psychological adaptation and is attracting a lot of attention in scientific fields, the neural basis of EC remains unclear. Furthermore, EC includes three key components: inhibitory control (IC), activation control (ACTC), and attentional control (ATC). IC is the ability of suppression the inappropriate behavior. ACTC is an ability to do somehing when there are higher tendencies to refrain from it. ATC is the mental ability of focusing and shifting attention when desired. Although there were positive correlation among three components (IC-ACTS: r = 0.48, IC-ATC: r = 0.42, and ACTC-ATC: r = 0.44)^9^, previous psychological studies demonstrated that three components of EC were associated with different psychological behavior. IC was correlated with performance of Stroop task^10^. IC and ACTC was negatively correlated with depression^9^. ACTC was positively correlated with conscientious personality^10^. ATC was negatively associated with social anxiety^9^. These results indicated that there would be different neural correlates in each sub-component of EC. However, no report has described a study directly investigating the relation between the brain structure and individual differences of three key components of EC: IC, ACTC, and ATC.

Based on previous psychological and neuroimaging evidence, we made the following hypotheses in each key component of EC. First, we hypothesized that individual differences of IC, which measure the capacity to inhibit inappropriate responses, would be associated with inhibition related brain areas like the dorsal anterior cingulate cortex (dACC), the supplementary motor area (SMA), and the inferior frontal gyrus (IFG), because the dACC, SMA, and IFG were activated during inhibit tasks^11^,^12^,^13^. Second, we assumed that the putamen and the insula would be associated with individual differences in ACTC, which measures the ability to perform some behavior when there is a strong tendency to refrain from doing something^10^,^14^,^15^. In other words, the ACTC score represents an ability to do one’s job properly. Previous psychological studies reported that the ACTC score had a strong correlation with the score for conscientious measured by the NEO-FFI personality score^10^. The concept of conscientious in NEO-FFI is characterized by orderliness, responsibility, and dependability^16^. Moreover, one recent neuroimaging study has demonstrated the score of the conscientious personality scale was negatively associated with the white matter volume in the putamen and the insula^17^. Therefore, we made the hypothesis which the putamen and the insula would be associated with individual differences in ACTC. Third, we hypothesized that individual differences in the ATC, which measure the individual differences of focus and shift attention, to ignore information, would be associated with attention related brain areasas the anterior part of insula (AI), the ACC and the dorsolateral prefrontal cortex (DLPFC), because these brain regions were activated during focus, shift, and divided attention tasks^18^,^19^,^20^.

To test these hypotheses, we investigated correlation between IC, ACTC, and ATC in EC and rGMV (regional gray matter volume) and rWMV by voxel-based morphometry (VBM)^21^. We used the the Effortful control scale in Japanese (J-ECS) to measure IC, ACTC, and ATC^10^,^14^.

## Results

### Basic data

The averages, standard deviations (SD), and Variance Inflation Factor (VIF) of age, each subscale of EC (IC, ATC, and ACTC), and Raven’s Advanced Progressive Matrix (RAPM) were shown in Table 1. A Pearson’s correlations coefficients between these variables were shown in Table 2. We confirmed good reliabilities of each subscale in EC using Cronbach’s alpha in our sample (IC = 0.805, ATC = 0.823, and ACTC = 0.812). We also confirmed the normal distributions of IC, ATC, and ACTC based on histograms and Quantile-Quantile (Q-Q) plots (see Supplementary Fig. S1).

### Correlations between rGMV/rWMV and IC

**Figure 1:**
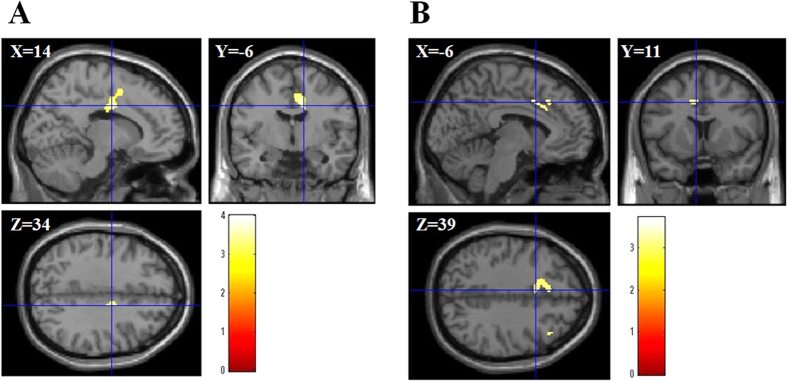
Anatomical bases of IC. (**A**) rGMV was positively associated with IC in areas in the right SMA and the right dACC (MNI coordinates of the peak, *x*, *y*, *z* = 14, −6, 34). Results are shown with *P* < 0.05 (F.W.E). The red bar shows the T score. (**B**) rWMV was positively associated with IC in areas in the left dACC (MNI coordinates of the peak, *x*, *y*, *z* = −6, 11, 39). The red bar shows the T score.

We investigated the correlation between rGMV/rWMV and IC score using total brain volume, age, RAPM, sex, ACTC, and ACT as covariates. The result showed the positive correlation between IC and rGMV in the right SMA, pre SMA, and right dorsal ACC (dACC) (Montreal Neurological Institute (MNI) coordinates of the peak, *x*, *y*, *z* = 14, −6, 34; *t* value = 4.00; corrected *P* value at the cluster = 0.045; Fig. 1A).

The multiple regression analysis for rWMV revealed that the IC score was positively correlated with rWMV in the left dACC (MNI coordinates of the peak, *x*, *y*, *z* = −6, 11, 39; *t* value = 3.37; corrected *P* value at the cluster = 0.004; Fig. 1B).

### Correlations between rGMV/rWMV and ACTC

**Figure 2:**
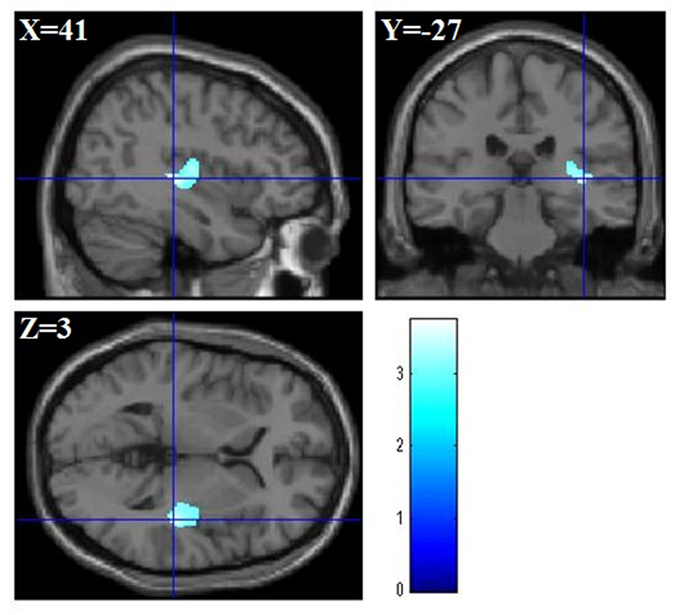
Anatomical bases of ACTC. rGMV was negatively associated with ACTC in areas in the right Heschl’s gyrus, the right putamen, and the right insula (MNI coordinates of the peak, *x*, *y*, *z* = 41, −27, 3). The blue bar shows the T score.

We investigated the correlation between rGMV/rWMV and ACTC score using total brain volume, age, RAPM, sex, IC, and ACT as covariates. The multiple regression analysis for rGMV showed that the ACTC was negatively associated with rGMV in the right Heschl’s gyrus, the right putamen, and the right insula (MNI coordinates of the peak, *x*, *y*, *z* = 41, −27, 3; *t* value = 3.74; corrected *P* value at the cluster = 0.007; Fig. 2). On the other hand, we did not find any significant correlations between the scores of ACTC and rWMV.

### Correlations between rGMV/rWMV and ATC

**Figure 3:**
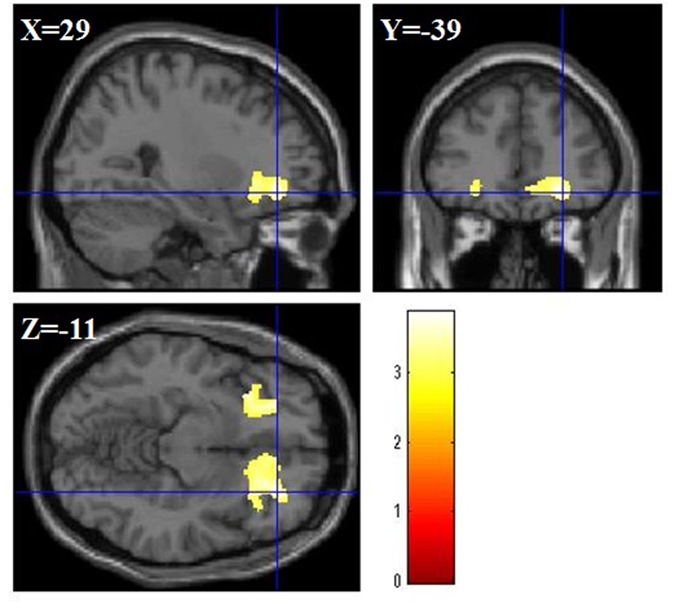
Anatomical bases of ATC. rWMV was positively asociated with individual ATC in a areas in the right IFG, the bilateral ACC, the bilateral OFC, and the bilateral insula (MNI coordinates of the peak, *x*, *y*, *z* = 29, 39, −11). The red bar shows the T score.

We investigated the correlation between rGMV/rWMV and ATC score using total brain volume, age, RAPM, sex, ACTC, and IC as covariates. The multiple regression analysis for rGMV using ATC showed no significant correlations between rGMV and ATC score. On the other hand, multiple regression analysis for rWMV using ATC score showed the positive correlation between ATC score and rWMV in the inferior frontal gyrus (IFG) in the right part, bilateral orbito frontal cortex (OFC), ACC, and insula (MNI coordinates of the peak, *x*, *y*, *z* = 29, 39, −11; *t* value = 3.87; corrected *P* value at the cluster < 0.001; Fig. 3).

## Discussion

In this study, we firstly showed the significant associations between rGMV/rWMV and EC scores. This study yielded three main findings for each subscale (IC, ACTC, and ATC). First, the IC score was collated with larger rGMV in the dACC and the SMA and larger rWMV in the dACC. Second, the ACTC score was associated with smaller rGMV in the insula and the putamen. Third, the ATC score was collated with larger rWMV in the right IFG, the bilateral OFC, the bilateral ACC, and the bilateral insula. These main findings are considered in more detail below.

Larger rGMV of in the dACC, the SMA, and the pre-SMA and larger rWMV in the dACC in participants with higher IC might be a reflection of the capacity to suppress inappropriate response or to inhibit response. The dACC, the pre- SMA, and the SMA are important for he inhibiting process. These regions are consistently activated during inhibit tasks like the Stroop task, the stop-signal task, and Go/No-Go task^11^,^22^,^23^. Additionally, recent anatomical study using VBM revealed the significant associations between the rGMV in the dACC and the Stroop task performance which measures the inhibition ability^24^. Our result is supported previous evidence which reported the significant associations between total score of EC and rGMV in the SMA^25^ and performance of inhibition task and gray matter density in the pre-SMA^26^. Previous studies using DTI showed significant correlation between Stroop performance and white matter integrity related dACC^27^. Consequently, more gray and white matter in these areas in people with higher IC may correlated with their higher capacity to inhibit response.

However, compared to the previous studies related to IC^11^,^22^,^23^, the peak of the dACC’s cluster of rGMV/rWMV in the present results was located more ventrally and more posteriorly. Looking at the items included IC, several items were related to inhibition behaviors in social situations (e.g. “I can easily resist talking out of turn, even when I’m excited and want to express an idea” or “When I see an attractive item in a store, it’s usually very hard for me to resist buying it”). The dACC has several functions related to emotion and cognition processes^28^. Previous study using diffusion-weighted imaging (DWI) tractography showed that the posterior dACC and the ventral region of dACC like the current study was more associated with emotion and social interaction^29^. Taken together, we found the significant correlation between IC score and the more ventral and posterior part of dACC, because IC measures inhibition behaviors related to social situations. In the future study we should investigate this assumption using social inhibition scales.

Although the right IFG is important for inhibition^30^, we found no significant correlations between IC and the right IFG. Recent neuroimaging studies using between-subject and within-subject approaches showed the greater activity in the SMA activation during shorter Stop-Signal reaction times people (sessions) compared to longer Stop-Signal reaction times people (sessions), but not in the right IFG^31^,^32^. Other recent research have suggested that the SMA is selectively associated with inhibitions of responses, whereas the right IFG activations are highly related to attentional capture^33^ and attentional control^34^. Supporting these previous findings, the present results showed a positive association between ACT and rWMV in the right part of IFG. Taken together, the right IFG may be important for attentional processes, but not for inhibition processes. However, these are speculations that must be examined in future studies to confirm these issues.

Smaller rGMV in the insula and the putamen in participants with higher ACTC would be correlated with an ability to reduce feeling of disgust and hating something. Therefore, it may induce characteristics of ACTC such as an ability to do one’s job properly and to have a responsible and conscientious personality. Previous studies suggested that the insula and putamen have a critical roles in the so-called hate circuit^35^. Previous neuroimaging studies demonstrated that the insula and putamen were significantly activated during viewing disgust face^36^. In addition, previous neuropsychological study showed that the insula and putamen damaged patient did not recognize disgust from multiple modalities (e.g. face, non-verbal emotional sound, and emotional prosody)^37^. Consequently, reduced rGMV in the insula and putamen may be correlated with a performance to reduce disgust feeling. Therefore, because of less ability to feel disgust or hate, people with smaller rGMV in these regions can do something which other people do not want to do. Finally, it may lead to characteristics of ACTC such as the ability to do one’s job properly and responsible and conscientious personality. We did not measure individual differences of disgust in the present study. Therefore, future studies must confirm our idea using measurements for sensitivity to disgust such as a disgust scale^38^.

Larger rWMV in the IFG, ACC and insula in participants with higher ATC may reflect the capacity of shifting and focusing attention. These regions were consistently activated during focus and selective attention tasks^18^,^19^,^39^. In addition, previous DTI studies showed significant associations between white matter integrities in the IFG, ACC, and insula and attentional tasks^40^,^41^. Intervention studies using mindfulness meditation called integrated body mind training showed the improvement of attention performance and the greater change in fractional anisotropy in the ACC^42^. Consequently, more white matter in these areas in people with higher ATC may associate with their higher capacity of shifting and focusing attention.

Our study did not find any significant associations between the ATC and rGMV/rWMV in the DLPFC. These non-significant results might be explained by the distinct roles of the DLPFC in attentional control. Previous neuroimaging studies demonstrated that the DLPFC was activated during situations that required dual-task performance^43^,^44^,^45^. The scores of the ATC in the J-ECS represent the ability of concentrating on one task (focusing attention) and alternately shifting from doing one thing to doing the other things (shifting attention)^10^,^14^. The score of the ATC in the J-ECS does not measure the ability to do two different things simultaneously (dual-task performance). Therefore, we did not find any significant associations between the score of ATC and the brain structure in the DLPFC. Of course, the DLPFC also has a critical role in attention control during single-tasking^46^. Looking at items of ATC, several items of ATC measure needs to emotional regulate and attentional shifting under emotional situations (“When I am happy and excited about an upcoming event, I have a hard time focusing my attention on tasks that require concentration” or “It is very hard for me to focus my attention when I am distressed”). Indeed, ATC was associated with emotional regulations such as a control of anger^47^. There is a possibility that the score of ATC in EC represents emotional aspects of attention control such as need of emotional regulation, but not cognitive aspects of attention control. Supporting this possibility, our result showed the significant correlation between ATC and ventromedial prefrontal cortex (VMPFC), which has a key role of emotional regulation^48^. In the future research, it is important to study the relationship among brain structure, cognitive aspects of attention control, and emotional aspects of attention control.

This study has some limitations First, we used a self-report questionnaire to measure EC. Self-reports questionnaires may be affected by some biases such as social desirability. Thus, it may not be possible to avoid that bias in the current study. In the future study to investigate neural bases of EC, it is important to use both a self-report questionnaire and behavior tasks which can measure the EC such as Stroop test and attentional tasks. Second, the current study used a sample of homologous young healthy adults, which may make the findings difficult to generalize to the wider populations. To generalize our findings, it needs to use other populations such as children and older adults. These are an important limitation in the current study.

## Conclusions

In summary, EC is a basis for individual differences in managements of emotions, thoughts, and actions. Additionally, EC plays a key part in psychological adjustment processes. However, the neural basis of EC remains unknown. Using VBM, we investigated associations between individual differences of rGMV/rWMV and the subscales of EC such as IC, ACTC, and ATC. We revealed that (a) the IC score was correlated with large rGMV in the dACC and the SMA and large rWMV in the dACC, (b) the ACTC score was correlated with smaller rGMVin the insula and the putamen, and (c) the ATC score was associated with larger rWMV in the IFG, OFC, and insula. These findings firstly demonstrated that a neuroanatomical correlation between rGMV/ rWMV and EC.

## Methods

### Subjects

Total right-handed 680 healthy students (374 men and 306 women; 20.61 ± 1.82 years) participated in this study. The methods for subjects were described in our previous study and reproduced below^49^. The participants were also participated in our ongoing project^49^,^50^. They had no histories of psychiatric and neurological diseases. The Edinburgh Handedness Inventory was used to evaluate participants’ handedness^51^. Based on the Declaration of Helsinki (1991), we obtained written informed consent from every subjects. This study was approved by the Ethics Committee of the Tohoku University Graduate School of Medicine. The methods of this study were also carried out in accordance with the approved guidelines.

### Effortful control scale (EC)

The J-ECS^10^,^14^, is a part of in the adult temperament questionnaire^15^, was used to measure subject’s effortful control. The scale has 35 items with a 4-points Likert scale). The EC scale consists of three subscales such as Attention control (ATC), Activation Control (ACTC), and Inhibitory Control (IC). Eleven items of IC measure an ability to inhibit inappropriate behavior (e.g., ‘When I decide to quit a habitual behavioral pattern that I believed to be undesirable, I am usually successful’^10^,^14^). Twelve items of ACTC measure the ability to to do somehing when there are higher tendencies to refrain from it (e.g., ‘I can make myself work on a difficult task even when I don’t feel like trying’^10^,^14^). Twelve items of ATC measure focusing and shifting attentional abilities (e.g., ‘When interrupted or distracted, I usually can easily shift my attention back to whatever I was doing before’^10^,^14^). The J-ECS of the 35 EC items from the Adult Temperament Questionnaire was developed through a back-translation procedure^15^. The J-ECS has good internal consistency such as Cronbach’s alpha were 0.74 (IC), 0.83 (ACTC), and 0.84 (ATC)^10^. Test–retest reliabilities were *r* = 0.79 (IC), *r* = 0.89 (ACTC), and *r* = 0.81 (ATC). Validity of EC was confirmed by significant associations between the performance of the Stroop task and the EC score^10^ and between the performance of the Go/No-Go task and the EC score^52^.

### Raven’s Advanced Progressive Matrix

General and basic intelligence was measured using Raven’s Advanced Progressive Matrix RAPM^53^. We also used RAPM score for adjustment for the effect of general intelligence on rGMV and rWMV^54^. Please see our previous studies^49^.

### Image acquisition and analysis

The methods for MR image acquisition were described in our previous study and reproduced below^49^. MRI data acquisition was performed using a 3-T Philips Achieva scanner. High-resolution T1-weighted structural images (T1WIs: 240 × 240 matrix, TR = 6.5 ms, TE = 3 ms, FOV = 24 cm, slices = 162, slice thickness = 1.0 mm) were collected using a magnetization-prepared rapid gradient echo sequence.

### Preprocessing of T1-weighted structural data

We analyzed all data by Statistical Parametric Mapping software (SPM8), Wellcome Department of Cognitive Neurology, London, UK and Matlab, Mathworks Inc., Natick, MA, USA. We used the new segmentation tool with the International Consortium for Brain Mapping (ICBM) template for East Asian brains. Then we used the DARTEL (diffeomorphic anatomical registration through exponentiated lie algebra) registration process withgray and white matter tissue probability maps (TPMs) created by the new segmentation process. First, the template of the DARTEL was created based on all 680 participants (374 men and 306 women). Using this existing template, DARTEL was performed for all participants. The resultant images were spatially normalized to MNI space to give images with 1.5 × 1.5 × 1.5 mm^3^ voxels.rGMV and rWMV were smoothed by convolving them with an isotropic Gaussian kernel of 10 mm FWHM (full width at half maximum).

To check whether our data were affected by artifacts or errors during segmentation and normalization process, we performed a sample homogeneity check by covariance check in the VBM8 toolbox for each smoothed rGMV an rWMV maps. In this test, the squared distance to the sample mean was calculated. We checked the quality of all data using the following steps. First, we checked whether the distance was larger than twice the sample standard deviation. Second if the squared distance was lartger than twice the standard deviation of the sample, we checked both the rGMV/rWMV maps and the original image were visually inspected. Finally, we found that, all GMV/WMV maps were eligible for a further analysis.

### Statistical analyses for imaging data

Whole-brain multiple regression analyses were conducted to investigate correlations between IC, ACTC, and ATC as measured by J-ECS and rGMV/rWMV. SPM8 was used analyses for imaging data. Each score of IC, ACTC, and ATC was included in the model of the multiple regression analysis in order to control of the effect of the other component of EC as covariates. The multiple regression used RAPM score, total intracranial volume (TIV; total GM volume + total WM volume + total CSF volume), age and sex as additional covariates. We used the “overall mean” option for centering. Based on previous studies^49^,^55^, we used only voxels in rGMV/rWMV that showed values of >0.05.

To check multicollinearity, we calculated Variance Inflation Factor (VIF) in the multiple regression analyses using the Design Magic toolbox (http://www.ni-utrecht.nl/downloads/d_magic). The Design Magic calculated the R-square for all factors of a design matrix generated in SPM. The VIF was calculated the following formula (VIF = 1/(1 − R-square)). The VIF values of between 1.02 and 1.55 were obtained. These results indicated that the multicollinearity did not exist because VIF values above 4 indicate multicollinearity problems^56^. Thus, we conducted the abovementioned regressions.

We set at *P* < 0.05 (FWE, Family-Wise Error) using non-stationary cluster-extent corrections^57^ based on voxel level of *P* < 0.0025. In the random field theory with non-isotropic cluster-size test, a cluster-determining threshold using high smoothing values of more than six voxels leads to appropriate conservativeness. We used *P* < 0.0025 because, an uncorrected threshold of *P* < 0.001 using high smooth values is slightly conservativeness^58^.

## Additional Information

**How to cite this article**: Nouchi, R. *et al*. Neuroanatomical bases of effortful control: evidence from a large sample of young healthy adults using voxel-based morphometry. *Sci. Rep.* **6**, 31231; doi: 10.1038/srep31231 (2016).

## Supplementary Material

Supplementary Information

## Figures and Tables

**Table 1:** Basic data of the participants.

Measure	Mean	SD	VIF
Age	20.61	1.83	1.02
RAPM	28.62	3.78	1.04
Inhibitory control (IC)	29.33	4.68	1.54
Activation control (ACTC)	30.52	5.80	1.39
Attentional control (ATC)	24.50	4.77	1.55

SD: standard deviation, RAPM: Raven’s Advanced Progressive Matrix, VIF: Variance Inflation Factor.

**Table 2:** A Spearman’s correlation matrix.

	Age	RAPM	Inhibitory control (IC)	Activation control (ACTC)	Attentional control (AC)
Age	1				
RAPM	−0.003	1			
Inhibitory control					
(IC)	0.122**	0.035	1		
Activation control					
(ACTC)	0.064	−0.086*	0.454**	1	
Attentional control					
(ATC)	0.132**	0.114**	0.537**	0.440**	1

*P < 0.05, **P < 0.01.
