# Supplementary material for: Neuroanatomical bases of effortful control: evidence from a large sample of young healthy adults using voxel-based morphometry
Source: Sci Rep. 2016 Aug 9;6:31231. doi: 10.1038/srep31231 (PMC4977574; doi:10.1038/srep31231)
Supplement: Supplementary Information [file srep31231-s1.pdf]

Neuroanatomical bases of effortful control: evidence from a large sample of young healthy adults using voxel-based morphometry

Rui Nouchi[a,b], Hikaru Takeuchi[c], Yasuyuki Taki[c,d,e], Atsushi Sekiguchi[e,f], Yuka Kotozaki[b], Seishu Nakagawa[g], Carlos Makoto Miyauchi[h], Kunio Iizuka[g,i], Ryoichi Yokoyama[j,k], Takamitsu Shinada[g], Yuki Yamamoto[g], Sugiko Hanawa[g], Tsuyoshi Araki[b], Yuko Sassa[c], Ryuta Kawashima[b, c, g]

[a]*Creative Interdisciplinary Research Division, Frontier Research Institute for Interdisciplinary Science （FRIS), Tohoku University, Seiryo-machi 4-1, Sendai 980-8575, Japan*

[b]*Smart Ageing International Research Center, Institute of Development, Aging and Cancer, Tohoku University, Sendai, Japan*

[c]*Division of Developmental Cognitive Neuroscience, Institute of Development, Aging and Cancer, Tohoku University, Sendai, Japan*

[d]*Department of Nuclear Medicine and Radiology, Institute of Development, Aging and Cancer, Tohoku University, Sendai, Japan*

[e]*Division of Medical Neuroimaging Analysis, Department of Community Medical Supports, Tohoku Medical Megabank Organization, Tohoku University, Sendai, Japan*

[f]*Department of Adult Mental Health, National Institute of Mental Health, National Center of Neurology and Psychiatry, 4-1-1 Ogawa-Higashi, Kodaira, Tokyo 187-8553, Japan*

[g]*Department of Functional Brain Imaging, Institute of Development, Aging and Cancer, Tohoku University, Sendai, Japan*

[h] *Graduate Schools for Law and Politics,　The University of Tokyo,　Tokyo,　Japan*

[i]*Department of Psychiatry, Tohoku University Graduate School of Medicine, Sendai, Japan*

[j] *Japan Society for the Promotion of Science, Tokyo, Japan*

[k] *Faculty of Medicine, Kobe University, 7-5-1kusunoki-cho, Kobe, 950-0017 Japan*

Corresponding author:

Rui Nouchi, Ph. D, Assistant Professor

Creative Interdisciplinary Research Division,

Frontier Research Institute for Interdisciplinary Science (FRIS), Tohoku University

Seiryo-machi 4-1, Aoba-ku, Sendai 980-8575, Japan

Tel/Fax: +81-22-717-7988

E-mail: rui.nouchi.a4@tohoku.ac.jp

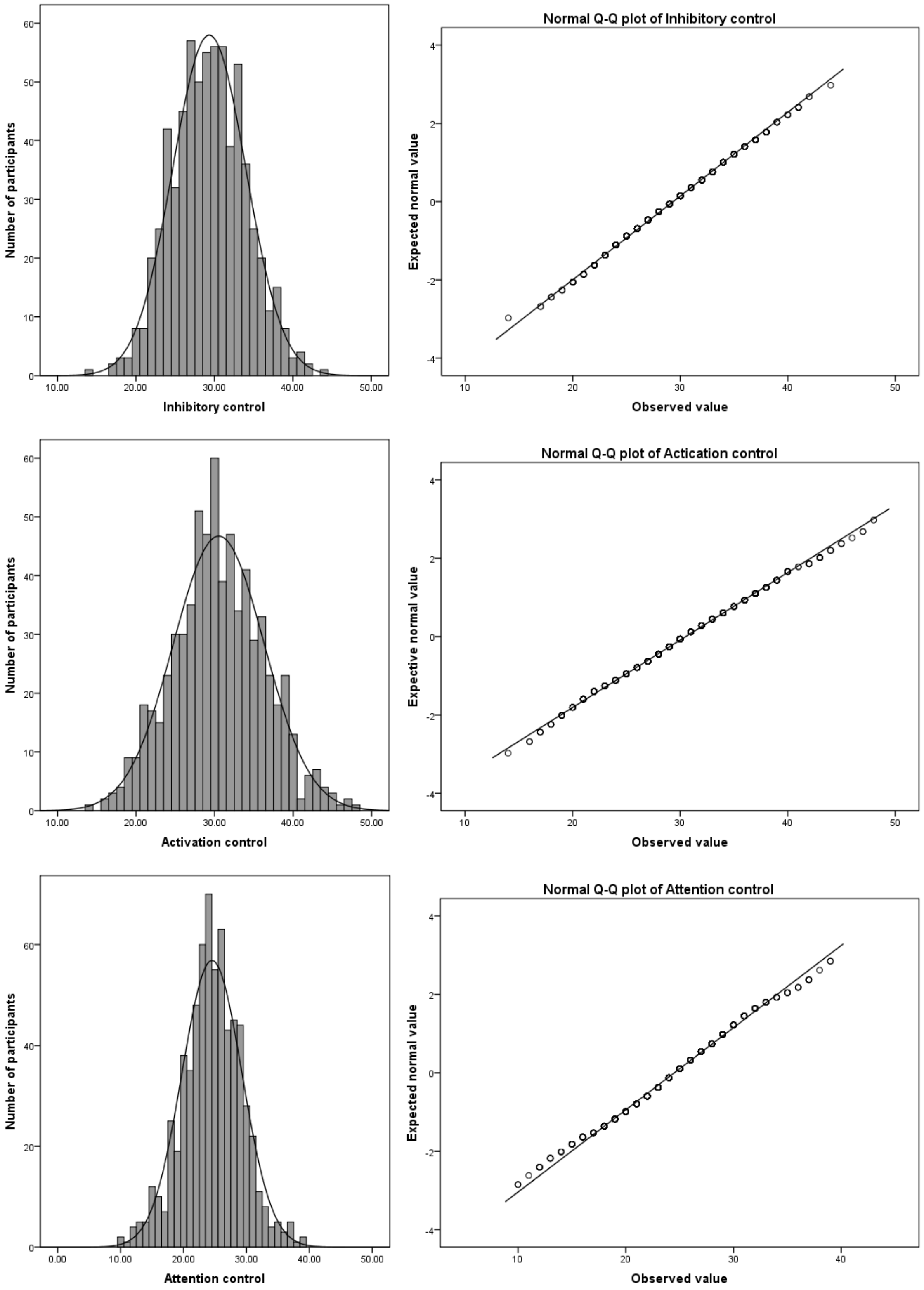

Figure S1. Histograms and Quantile-Quantile in inhibitory control (top), actication control (middle), and attention control (bottom).
